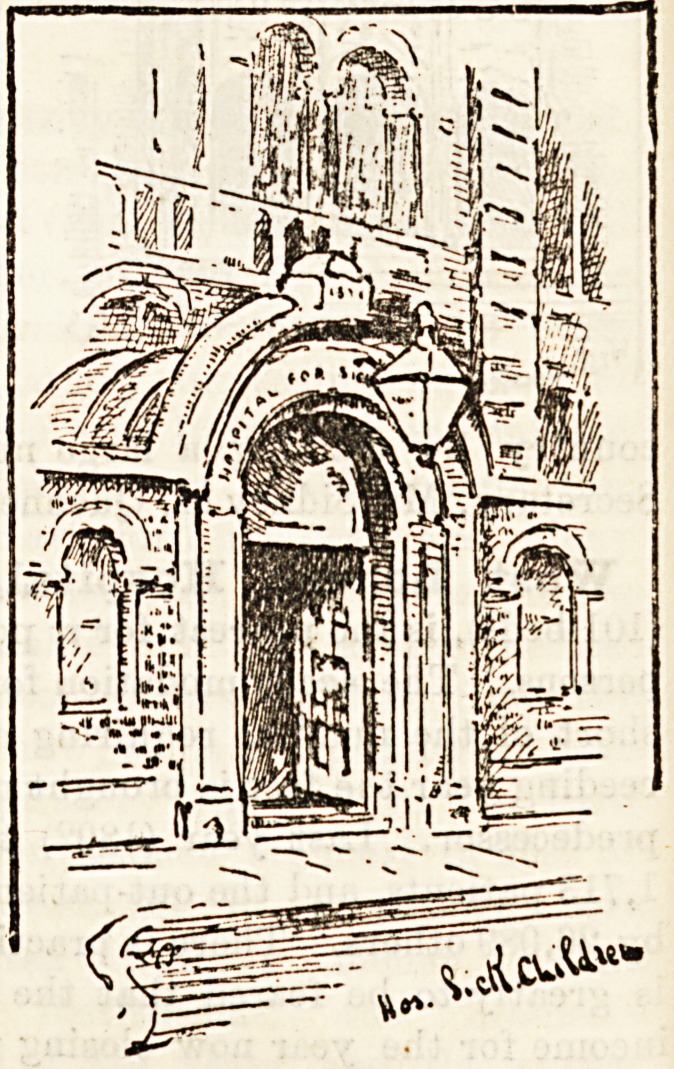# Children

**Published:** 1893-12-23

**Authors:** 


### CHILDREN.

**East London Hospital for Children,** Shadwell, E. —An excellent institution, the original home of which in Ratcliff Highway was graphically described by Charles Dickens, whose memorable account will live for generations. Unfortunately, the children admitted are in as dire straits now as then, and funds are sadly needed. Secretary, Mr. Samuel Whitford.

**North-Eastern Hospital for Children,** Hackney Road, N.E.—This hospital seems to have been most unjustly neglected by the charitable public for some time past. During the last three years it has had a deficit of over £1,000 a-year, and it is now £4,500 in debt. This state of things is, we believe, partly due to its being hidden away from the gaze of the well-to-do classes in the midst of the poorest districts of London, and partly to the fact that it is too poor to spend the money necessary to bring its work properly before the public, who, however, we are sure, will at once come forward with substantial help when they learn that the hospital has been brought to such straits as to leave the committee no alternative but to reduce the number of patients by half unless £1,000 can be got together within the next month. The average number of out-patients annually is over 14,000, and of in-patients over 700. Secretary, T. Glenton-Kerr; Matron, Miss E. W. Curno; City office, 27, Clement's Lane, E.C.; Bankers, Barclay, Bevan, and Co.

**Paddington Green Children's Hospital,** W.—After many years of work in two houses adapted to the purposes of a hospital, outbreaks of illness rendered it increasingly evident that the structure was absolutely unfit, and the fact was forced upon the committee that rebuilding was the only remedy. At least £12,500 will be required for a plain, but efficient hospital; £7,000 are in hand, and the committee earnestly desire to make the charity more known, and so plead for help from the benevolent for the sum of £5,500 still required. No other hospital exclusively devoted to children exists in the parish of Paddington, or in the adjoining one of Marylebone. It has, therefore, special claims, and the committee trust that there will be a liberal response to their appeal. The smallest donation will be most thankfully received. Secretary, Mr. W. H. Pearce; Matron, Miss Anderson.

**The Hospital for Sick Children,** Great Ormond Street, London, W.C.—Founded in 1852, with ten beds, this was the first hospital solely devoted to the sick children of the poor. There are now nearly 200 beds at Great Ormond Street, besides 52 beds at Highgate. Over 1,600 in-patients, besides about 22,000 new out-patients, have been treated in 1893 at a cost of £15,000. The financial condition of this great charity is desperate. With a debt of £9,000 incurred in finishing the new wing, the committee have been obliged to borrow £3,000 to pay for the cost of the last three months maintenance. Unless this sum of £12,000 can be raised in the next six months the entire hospital will probably have to be closed. Surely the rich and charitable public will step forward to prevent such a calamity. Secretary, Adrian Hope; Lady Superintendent, Miss Close.

**Victoria Hospital for Children,** Queen's Road, Chelsea.—Established at Chelsea in 1866 for the relief of the sick and suffering children of the poor, this unendowed hospital seeks further subscriptions to help it to carry on its work. The expenditure, which is only 19s. per patient, yet reaches £5,000 to £6,000 a year, and this has to be raised entirely by voluntary subscriptions. Secretary, Commander W. C. Blount, R.N.; Matron, Miss Cooper.

## Figures and Tables

**Figure f1:**